# Prospective Cohort Study of the Kinetics of Specific Antibodies to SARS-CoV-2 Infection and to Four SARS-CoV-2 Vaccines Available in Serbia, and Vaccine Effectiveness: A 3-Month Interim Report

**DOI:** 10.3390/vaccines9091031

**Published:** 2021-09-17

**Authors:** Olivera Lijeskić, Ivana Klun, Marija Stamenov Djaković, Nenad Gligorić, Tijana Štajner, Jelena Srbljanović, Olgica Djurković-Djaković

**Affiliations:** 1 Department of Microbiology and Parasitology, Institute for Medical Research, University of Belgrade, 11129 Belgrade, Serbia; olivera.lijeskic@imi.bg.ac.rs (O.L.); iklun@imi.bg.ac.rs (I.K.); marija.stamenov@imi.bg.ac.rs (M.S.D.); tijana.stajner@imi.bg.ac.rs (T.Š.); jelena.srbljanovic@imi.bg.ac.rs (J.S.); 2 Faculty of Information Technology, Alfa BK University, 11070 Belgrade, Serbia; office@zentrixlab.eu; 3 Zentrix Lab, 26000 Pančevo, Serbia

**Keywords:** SARS-CoV-2, COVID-19, BNT-162b2, BBIBP-CorV, Gam-COVID-Vac, ChAdOx1-S, specific antibodies, vaccine effectiveness

## Abstract

Real-life data on the performance of vaccines against SARS-CoV-2 are still limited. We here present the rates of detection and levels of antibodies specific for the SARS-CoV-2 spike protein RBD (receptor binding domain) elicited by four vaccines available in Serbia, including BNT-162b2 (BioNTech/Pfizer), BBIBP-CorV (Sinopharm), Gam-COVID-Vac (Gamaleya Research Institute) and ChAdOx1-S (AstraZeneca), compared with those after documented COVID-19, at 6 weeks and 3 months post first vaccine dose or post-infection. Six weeks post first vaccine dose, specific IgG antibodies were detected in 100% of individuals fully vaccinated with BNT-162b2 (*n* = 100) and Gam-COVID-Vac (*n* = 12) and in 81.7% of BBIBP-CorV recipients (*n* = 148), while one dose of ChAdOx1-S (*n* = 24) induced specific antibodies in 75%. Antibody levels elicited by BNT-162b2 were higher, while those elicited by BBIBP-CorV were lower, than after SARS-CoV-2 infection. By 3 months post-vaccination, antibody levels decreased but remained ≥20-fold above the cut-off in BNT-162b2 but not in BBIBP-CorV recipients, when an additional 30% were seronegative. For all vaccines, antibody levels were higher in individuals with past COVID-19 than in naïve individuals. A total of twelve new infections occurred within the first 3 months post-vaccination, eight after the first dose of BNT-162b2 and ChAdOx1-S (one each) and BBIBP-CorV (six), and four after full vaccination with BBIBP-CorV, but none required hospitalization.

## 1. Introduction

Since the beginning of the coronavirus disease 2019 (COVID-19) pandemic, more than 170 M cases have been recorded to date (3 June 2021), claiming more than 3.5 M lives, and disrupting life at a global level. This tremendous challenge was counteracted by an unprecedented effort to develop, scale-up production and distribute vaccines against severe acute respiratory syndrome coronavirus 2 (SARS-CoV-2) on a global scale. Of the 17 vaccines approved for emergency or full use by diverse national regulatory authorities so far, 6 have been approved by at least one WHO-recognized stringent regulator [1]. Real-life studies of vaccines against SARS-CoV-2 are very much needed yet still extremely scarce [2,3,4,5,6].

Serbia is in a privileged position to monitor the effects of SARS-CoV-2 vaccines as four approved ones including the BNT-162b2 (BioNTech/Pfizer) mRNA vaccine, the BBIBP-CorV (Sinopharm), inactivated whole virus vaccine, and the vector vaccines Gam-COVID-Vac (Gamaleya Research Institute) and ChAdOx1-S (AstraZeneca), have been available (free of charge) to its citizens as of early 2021, of which the first three have been available since January, and ChAdOx1-S since late February. Although the vaccination rate has not been as good as desired, a total of 33.1% of citizens have been vaccinated so far [7].

In an attempt to understand the longevity of the humoral immune response induced by both natural infection and by the different vaccines, we have been carrying out a prospective study of the kinetics of IgG and IgM antibodies specific for SARS-CoV-2 by longitudinal follow-up of individuals with documented past COVID-19 (naturally immunized, NI) since August 2020, and of vaccinated individuals since January 2021.

We here present a first interim report on the rate of detection and level of specific antibodies to SARS-CoV-2, after natural infection and after vaccination at 6 weeks and 3 months post-infection and post-vaccination, as well as on the vaccine effectiveness at 3 months after administration of the first vaccine dose.

## 2. Materials and Methods

### 2.1. Study Design

A single-center prospective open cohort study of the long-term kinetics of SARS-CoV-2-specific antibodies after COVID-19 was initiated at the Institute for Medical Research in Belgrade in August 2020, by recruiting Institute employees and their family members, as well as their friends and colleagues, all with past COVID-19 confirmed by PCR/antigen test. As the vaccination campaign against SARS-CoV-2 in Serbia started in early January 2021, the study was broadened to include examination of vaccinated individuals. The study protocol includes examination of participants in both arms at fixed time points, specifically at 6 weeks, 3, 6, 9, 12 and 18 months (with an option to continue the study to 24 months and beyond) after infection or vaccination. The study is still recruiting participants.

### 2.2. Patients

A total of 110 adults (>18 years of age) with documented past COVID-19 were included in the naturally acquired immunity arm of the study. The vaccinal immunity arm included a total of 285 adults (of which 47 with documented past COVID-19), vaccinated with any of the four vaccines (BNT-162b2, BBIBP-CorV, Gam-COVID-Vac and ChAdOx1-S) available in Serbia.

For both groups, willingness to participate (informed consent) and commitment to coming back at fixed time points for the duration of the study were inclusion criteria. Participants in the natural immunity (NI) arm were excluded if and when vaccinated. 

Although we attempted to strictly adhere to the study protocol, some participants across all subgroups did not respect the exact follow-up time (but would come at a later point in time); for all here-reported calculations, only those examined at the time points of 6 weeks (39.12 ± 11.69 days for the NI group and 44.04 ± 8.26 days for the vaccinal group) and 3 months (86.77 ± 14.29 days for the NI group and 90.29 ± 9.78 days for the vaccinal group) were taken into account. 

### 2.3. Data Collection

At the time of presentation at the lab for blood drawing, all participants were interviewed. Basic demographic data (sex and age) were collected from all. For individuals who have had COVID-19, data on the clinical presentation of the disease were collected, including time of symptom onset (date of symptom onset was taken as day 1), self-reported symptoms and hospitalization. As we did not have access to their clinical files, to avoid imprecisions and potential bias, the severity of clinical presentation was based only on whether they were hospitalized or not, where hospitalization was taken as the measure of clinically serious disease. For vaccinated individuals, we collected data on the adverse reactions to the vaccines, and to assess vaccine effectiveness data on the occurrence of COVID-19 symptoms associated with a positive PCR/antigen test at any time post-vaccination were collected. Any individual who has experienced COVID-19 at any time after vaccination (either dose) with any vaccine was excluded from the calculations of the specific antibody response to the vaccine.

The study followed the principles of the Declaration of Helsinki. The protocol has been approved by a local (Institute for Medical Research) Ethics Committee (approval no. EO138/21).

### 2.4. Procedures

Blood samples were collected into serum tubes (Vacuette CAT Serum Clot Activator, Greiner Bio-One, Kremsmünster, Austria), according to standard operating procedures, centrifuged for 10 min at 900× *g*, and serum fraction was either immediately analyzed or stored at −20 °C until use. After analysis, the unused parts of all serum samples were placed in a −80 °C freezer for permanent storage. 

IgG and IgM antibodies specific for the SARS-CoV-2 spike protein receptor-binding domain (RBD) were detected by commercial bioMérieux tests (9COM and 9COG) on the fully automated VIDAS platform. These tests use a two-step sandwich enzyme immunoassay method with a final fluorescence detection (ELFA). The results are expressed as an index with a cut-off set at 1, indicating all findings <1 as negative, and ≥1 as positive. 

The specificity and positive percent agreement of the 9COG test have been evaluated to be ≥99% and 100% at ≥16 days of symptoms onset [8].

### 2.5. Statistical Analysis

To streamline the data collection, assessment, and analysis, patient data were stored in a relational database of the laboratory information system. In accordance with the EU General Data Protection Regulation 2018 and the according Serbian legislation (“Zakon o zaštiti podataka o ličnosti” of November 2018 (“Sl. glasnik RS“ br. 87)), data anonymization techniques have been implemented, and data were only stored on a local server behind the firewall. 

Descriptive statistics was used to analyze the basic characteristics of the patients; these data were analyzed as dichotomous variables (sex M/F, age ≤ and >65, hospitalization yes/no). Dichotomous variables were analyzed by χ2 or Fischer’s exact test as applicable. Differences in the IgG antibody kinetics (a) between different time points for each vaccine, (b) between different vaccines at same time points and (c) between any vaccine and NI individuals, were analyzed by Student’s *t*-test or Mann–Whitney test, for normal and non-normal distribution, respectively, as applicable. One-way Welch’s ANOVA was used to analyze differences in the antibody kinetics among all vaccines, followed by Games-Howell or Dunnett’s multiple comparison tests, depending on sample size. The level of significance was 0.05.

All statistics was performed with GraphPad Prism version 8.0.0 for Windows, GraphPad Software, San Diego, CA, USA, www.graphpad.com.

## 3. Results

### 3.1. Patients’ Characteristics

The basic characteristics of the patients in both arms of the study, at group level and per vaccine, are presented in Table 1. More than half of all participants (52%) were vaccinated with BBIBP-CorV, which vaguely reflects the general vaccinal situation in the country, as the BBIBP-CorV share among all vaccines is above 60% in Belgrade [9] and 70% in the country (by 21 April) [10]. On the other hand, there is an overrepresentation of BNT-162b2 (38% in our group vs. 14% in the country (by 21 April) [10], mainly because it was the one offered to willing Institute personnel.

Both the NI and the vaccinated groups, as a whole and per vaccine, were homogenous sex-wise. The BNT-162b2 group included more females than males, reflecting the female/male ratio in the Institute, but the difference compared with the NI group was not significant (*p* = 0.13). 

Age-wise, the share of individuals above 65 years of age was significantly (*p* = 0.04) larger in the BBIBP-CorV group than in the NI group, and in all other vaccine groups (vs. BNT-162b2 *p* < 0.001; vs. Gam-COVID-Vac *p* = 0.005, vs. ChAdOx1-S *p* = 0.003). This reflects the fact that at the beginning of the vaccination campaign BBIBP-CorV was the first one offered on a large-scale, and the elderly as a vulnerable yet responsible population were the first to respond. In contrast, the share of individuals vaccinated with BNT-162b2 below 65 years of age was larger than in the NI group (*p* = 0.001). 

Importantly, among the vaccinees, a subgroup of COVID-19-naïve individuals had been tested prior to vaccination. None of these were positive for SARS-CoV-2-specific IgG antibodies, and the mean antibody levels detected were at least an order of magnitude below cut-off (Table 1B). 

### 3.2. Levels of Specific IgG Antibodies in Non-Vaccinated Individuals with Past COVID-19 

**Figure 1:**
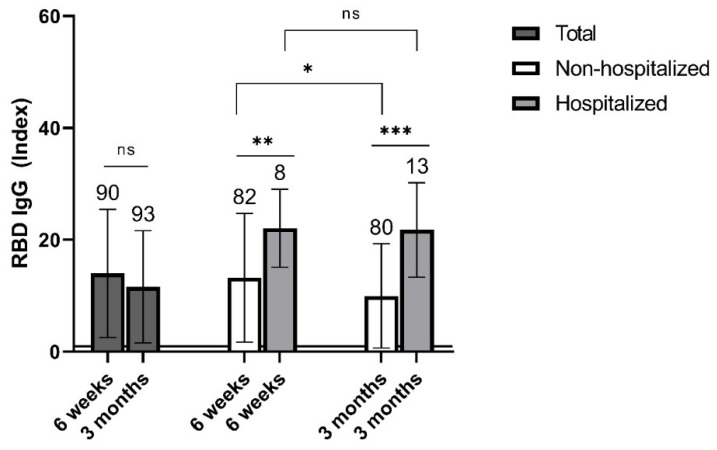
Mean levels of specific RBD IgG antibodies for individuals with past COVID-19 at 6 weeks and 3 months post infection. Results are presented for the whole group and according to hospitalization. Sample sizes are shown above standard deviation (SD) error bars. Horizontal line at an index of 1 indicates test cut-off. * *p* ≤ 0.05; ** *p* ≤ 0.01; *** *p* ≤ 0.001; ns—non-significant.

At 6 weeks post infection (p.i.), specific IgG antibodies were detected in all individuals examined at this time. By 3 months p.i., a non-significant (*p* = 0.12) decrease in antibody levels was registered (Figure 1). Moreover, three individuals reverted to seronegativity (data not shown). Importantly, when the group was stratified according to whether the participants were hospitalized or not for their past COVID-19 disease, IgG antibody levels were significantly higher in the hospitalized than in the non-hospitalized individuals at both time points (*p* = 0.005 and *p* < 0.001, at 6 weeks and 3 months p.i., respectively; Figure 1). Additionally, the antibody levels decreased significantly (*p* = 0.04) by 3 months p.i. in the non-hospitalized but not (*p* = 0.94) in the hospitalized individuals (Figure 1). Age distribution between the hospitalized and the non-hospitalized group was very similar (*p* = 0.9; data not shown).

### 3.3. Rate and Levels of Specific IgG Antibodies at 6 Weeks Post First Vaccine Dose 

Six weeks after the first vaccine dose (at the time when recipients of BNT-162b2, BBIBP-CorV, and Gam-COVID-Vac are considered to be fully vaccinated (as per manufacturers’ directions)), IgG antibodies were detected in 100% of those who received BNT-162b2 and Gam-COVID-Vac, and in 81.7% (94/115) of those who received BBIBP-CorV. Interestingly, specific IgG antibodies were detected in 75% (18/24) of individuals after one dose of ChAdOx1-S (data not shown).

**Figure 2:**
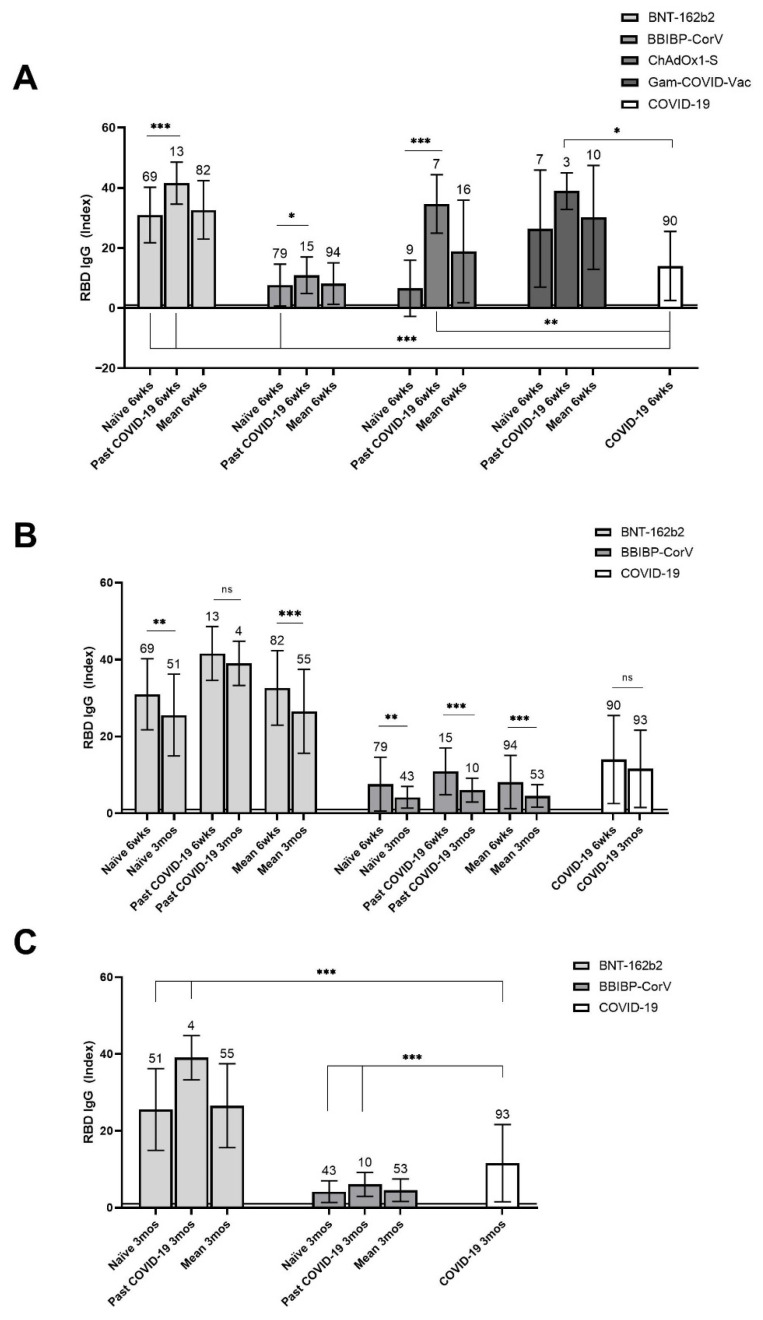
Mean levels of SARS-CoV-2 spike protein RBD-specific IgG antibodies post vaccination according to pre-vaccinal COVID-19 status, and post infection. (**A**)—in recipients of BNT-162b2, BBIBP-CorV, ChAdOx1-S, and Gam-COVID-Vac vaccines at 6 weeks post vaccination, as compared with non-vaccinated individuals with past COVID-19 at 6 weeks post infection. (**B**)—kinetics of antibody levels in recipients of BNT-162b2 and BBIBP-CorV from 6 weeks to 3 months post-vaccination, and in non-vaccinated individuals with past COVID-19 from 6 weeks to 3 months post infection. (**C**)—in recipients of BNT-162b2 and BBIBP-CorV at 3 months post vaccination, as compared with non-vaccinated individuals with past COVID-19 at 3 months post infection. (**A**–**C**)—Sample sizes are shown above standard deviation (SD) error bars. Horizontal line at an index of 1 indicates test cut-off. * *p* ≤ 0.05; ** *p* ≤ 0.01; *** *p* ≤ 0.001; ns—non-significant; wks—weeks; mos—months.

The level of specific IgG antibodies differed largely among the vaccinal groups (Figure 2A). Compared with the NI group, the mean antibody levels elicited by BNT-162b2 were significantly (*p* < 0.001) higher among both naïve and previously infected vaccinees. Mean antibody levels were also higher after the first dose of ChAdOx1-S and Gam-COVID-Vac in previously infected (*p* = 0.003, *p* = 0.03, respectively) but not in naïve individuals, when compared with the NI group. In contrast, IgG levels were significantly (*p* < 0.001) lower in naïve BBIBP-CorV vaccinees (Figure 2A). The lower levels in BBIBP-CorV recipients with past COVID-19 was not associated with the time elapsed after infection and the antibody decay in the interim [11,12], since the interval between infection and vaccination was not longer for BBIBP-CorV than for the other vaccines (*p* = 0.16; Table 1B).

A vaccinal booster effect was demonstrated for BNT-162b2, BBIBP-CorV and ChAdOx1-S, since at 6 weeks, vaccinees with past COVID-19 had significantly higher antibody levels than naïve participants vaccinated with these vaccines (*p* < 0.001, *p* = 0.02, *p* = 0.001, respectively; Figure 2A); for Gam-COVID-Vac this could not be demonstrated due to the small number of participants per subgroup.

### 3.4. Rate of Detection and Levels of Specific Antibodies at 3 Months Post First Vaccine Dose 

At the 3-month point, all recipients of BNT-162b2 and Gam-COVID-Vac still had specific IgG antibodies; however, among the recipients of BBIBP-CorV IgG, antibodies could no longer be detected in 30.3% (23/76). Vaccinees who became seronegative had low initial antibody levels (2.58 ± 0.87 at the 6-week time point), 

Antibody levels at 6 weeks and 3 months post-vaccination were compared in the BNT-162b2 and BBIBP-CorV groups (Figure 2B) (not for ChAdOx1-S because they had not yet reached this time point at the cutoff date of this study, nor for Gam-COVID-Vac where there were too few participants). The mean IgG levels significantly decreased in both vaccine groups (*p* < 0.001, *p* = 0.001, respectively, for BNT-162b2 and BBIBP-CorV), but remained ≥20-fold above cut-off. When compared with the NI group at 3 months, antibody levels for both previously infected and naïve vaccinees were significantly higher (*p* = 0.002, *p* < 0.001) in the BNT162b2, and lower (*p* < 0.001, *p* < 0.001) in the BBIBP-CorV group (Figure 2C). 

**Figure 3:**
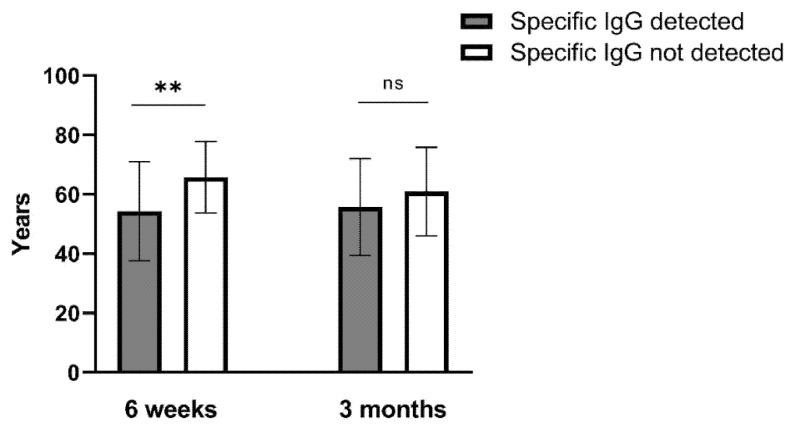
Age (mean ± SD) of BBIBP-CorV recipients according to the presence of SARS-CoV-2-specific IgG antibodies at 6 weeks and 3 months post vaccination. ** *p* ≤ 0.01; ns—non-significant.

Among the BBIBP-CorV recipients, individuals who did not develop antibodies were older than those who did. This difference was significant (*p* = 0.005) 6 weeks post vaccination (65.81 ± 12.09 vs. 54.31 ± 16.68, respectively), but not (*p* = 0.019) at 3 months post vaccination, when the mean age of those seronegative was 60.96 ± 14.93 vs. 55.72 ± 16.36 of those who remained seropositive (Figure 3). 

### 3.5. Analysis of the Vaccinal-Specific Antibody Levels According to Age

Vaccinal groups were stratified according to age into subgroups of individuals younger than 50, individuals between 50 and 65 years of age, and individuals older than 65. As stated above, since sample sizes for both Gam-COVID-Vac and ChAdOx1-S vaccinal groups were small, comparisons according to the different age strata were only performed for BNT-162b and BBIBP-CorV. No significant difference was found among the age strata in either the BNT-162b2 or the BBIBP-CorV vaccinal group at either time point (*p* = 0.23, *p* = 0.91, respectively, at 6 weeks post vaccination, and *p* = 0.69, *p* = 0.165, respectively, at 3 months post vaccination).

**Figure 4:**
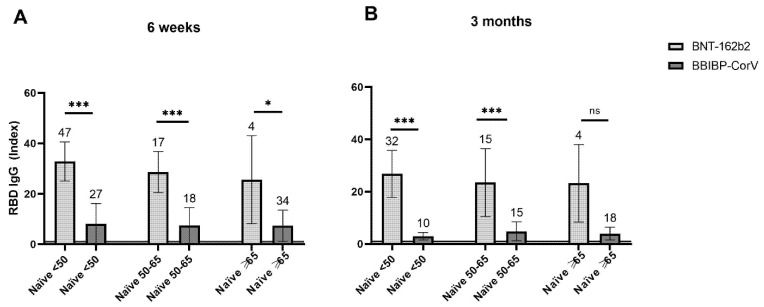
Mean levels of SARS-CoV-2 spike protein RBD-specific IgG antibodies post vaccination in naïve recipients of BNT-162b2 and BBIBP-CorV according to age (<50, 50–65, ≥65). (**A**)—at 6 weeks post vaccination, (**B**)—at 3 months post vaccination. (**A**,**B**)—Sample sizes are shown above standard deviation (SD) error bars. Horizontal line at an index of 1 indicates test cut-off. * *p* ≤ 0.05; *** *p* ≤ 0.001; ns—non-significant.

We further compared the groups of naïve BNT-162b2 and BBIBP-CorV vaccinees, stratified according to age, at 6 weeks and 3 months post vaccination. At both time points, in groups of individuals younger than 50 and 50–65 years old, antibody levels were significantly higher (*p* < 0.0001) in recipients of BNT-162b2 (Figure 4). This was also the case for participants older than 65 years of age at 6 weeks post vaccination (*p* = 0.012) (Figure 4A). However, the difference in the antibody levels was not significant (*p* = 0.065) among individuals older than 65 at 3 months post vaccination, likely due to a small sample and greater standard deviation in BNT-162b2 group (Figure 4B). 

### 3.6. Detection of Specific IgM Antibodies 

The rate of detection of specific IgM antibodies is presented in Table 2. Compared with SARS-CoV-2 infection, specific IgM antibodies were detected significantly less frequently in all vaccinal groups, and they decreased at a much higher rate by 3 months post vaccination than after SARS-CoV-2 infection (*p* < 0.001).

### 3.7. Vaccine Safety and Effectiveness 

As to the safety of the vaccines, other than immediate and usual reactogenicity, no adverse reactions to any of the vaccines were reported that would require medical assistance or reporting to the relevant national authority (Medicines and Medical Devices Agency of Serbia). 

Most importantly, the effectiveness of all vaccines within the first 3 months post-vaccination proved very high in the studied group. A total of 12 new infections were recorded during this timeframe, of which eight occurred after the first dose, of BBIBP-CorV in six cases, and of BNT-162b2 and ChAdOx1-S in one case each. Among fully vaccinated individuals, only four documented new infections were reported, all in those vaccinated with BBIBP-CorV (4/142, 2.8%). Importantly, none of the new infections, including the four breakthrough ones, required hospitalization.

## 4. Discussion

To the best of our knowledge, this is the first study that compares the real-life performance of four different vaccines against SARS-CoV-2. Although small in the number of participants, this study has yielded some important results: (a) An excellent effectiveness of all examined vaccines ranging between 97.2% and 100% up to three months post vaccination (for ChAdOx1-S up to 6 weeks). Breakthrough infections (a total of four) occurred only after vaccination with BBIBP-CorV, but none of these were severe. We do not have information on the viral variants involved in these new infections, i.e., whether they involved variants of concern; however, the only data published so far are from the June–October 2020 period, when different sub-lineages of the Alpha strain were detected in Serbia, most notably B.1.1.1 and B.1.1.70 [13]; (b) BNT-162b2 was able to protect 100% of fully vaccinated participants against symptomatic infection to 3 months post vaccination. A major Israeli study on 4,714,932 vaccinees has shown an effectiveness of 95.3% [2]; (c) Although BBIBP-CorV induced a comparably lower humoral immune response, as 18.3% recipients never developed IgG antibodies, and another 30% no longer had detectable antibodies at 3 months post-vaccination, even 97% were protected against symptomatic COVID-19 during this time. How well the specific antibody levels correlate with protection against COVID-19 is a matter of discussion [14]. Still, it may be expected that the latter individuals would be protected at least in part since they had detectable specific IgG antibodies at 6 weeks post vaccination, and therefore presence of memory B (and possibly T) cells may be assumed. This is less plausible for those who had never developed any specific antibodies. Moreover, this may be a particular concern for the elderly population, since individuals who never developed antibodies and those who became seronegative by 3 months tended to be older. 

Longitudinal follow-up of non-vaccinated individuals with past COVID-19 revealed that specific IgG antibody levels correlate with the clinical severity of the disease at both 6 weeks and 3 months p.i., being higher in hospitalized vs. non-hospitalized patients. A systematic review found specific antibody responses to frequently be associated with disease severity [15]. Moreover, a recent study based on a number of different specific antibody assays showed that asymptomatic individuals had the lowest responses, hospitalized individuals had the highest and symptomatic but not hospitalized individuals had intermediate-specific antibody responses [16]. 

The specific antibody levels induced by BNT-162b2 were higher, but by BBIBP-CorV were lower, at both time points here reported. Higher vaccinal than infection-induced antibody levels have been shown for mRNA vaccines (both BNT-162b2 and mRNA-1273), and Gam-COVID-Vac [17,18,19,20]. Additionally, vaccinal antibody levels were higher in previously infected than in naïve individuals (of note, this increase was the least with BBIBP-CorV).

An interesting observation concerns the difference in the detection of specific IgM antibodies after infection and vaccination. While detected in every three out of four non-vaccinated individuals with past COVID-19, in vaccinated individuals the rate of detection was remarkably lower at 6 weeks, and the elimination rate several-fold higher by 3 months after vaccination than after infection. The clinical significance of this, if any, is as yet unclear.

Vaccinal levels of specific antibodies higher than after SARS-CoV-2 infection suggest long-term protection. In contrast, the lower rate of detection of specific antibodies elicited by the BBIBP-CorV vaccine, their initial levels that are lower than in the NI group and compared with the other vaccines, all indicate a comparably lower immunogenicity of BBIBP-CorV. Similar results have already been obtained in the United Arab Emirates and Bahrain, where a third shot of this or another vaccine have been suggested [21]. Analysis according to age showed significantly lower mean antibody levels in vaccinees below 65 years of age who received BBIBP-CorV when compared with BNT-162b2 at both time points, as well as in those older than 65 at 6 weeks post vaccination. Interestingly, this difference was not significant in the oldest age group three months post vaccination, arguably due to small size of these age groups. However, it is reassuring that at least in this small study (at this early interim point) the BBIBP-CorV vaccine has proven highly effective in preventing serious infections.

The fact that this work was based on a serological assay rather than on a virus neutralization assay may be considered as a limitation. However, RBD-specific antibodies have been shown to be responsible for most (>90%) of the neutralizing antibody activity [22,23,24]. Peluso et al. [15] have recently shown high correlation between assays measuring responses to the spike protein and pseudovirus neutralization. Additionally, assessment of the vaccine effectiveness was based on symptomatic disease confirmed by PCR or antigen test, leaving the possibility of more mild infections that were not laboratory confirmed. Even if so, effectiveness against serious disease is beyond question. The higher than reported [2,5] effectiveness may in part be attributed to the higher education level of our study population, and consequently better compliance with prevention measures.

Presented here are early interim results of an ongoing study, at a time when ChAdOx1-S was not evaluated in fully vaccinated individuals. This study is ongoing, and will include an evaluation of ChAdOx1-S in fully vaccinated individuals in the next interim report.

## Figures and Tables

**Table 1:** Characteristics of the study group participants.

**A.** Unvaccinated participants with past COVID-19.									
	**Participants N (%)**	**Sex**		**Age**		**Hospitalization**			
		**F (%)**	**M (%)**	**≤65 (%)**	**>65 (%)**	**Yes (%)**		**No (%)**	
Past COVID-19 Unvaccinated	110	59 (53.6)	51 (46.4)	81 (73.6)	29 (26.4)	17 (15.5)		93 (84.5)	
**B.** Vaccinated participants.									
**Vaccinated Per Vaccine**	**Participants N (%)**	**Sex**		**Age**		**COVID-19 Naïve Tested Prior to Vaccination**		**Past COVID-19**	
		**F (%)**	**M (%)**	**≤65 (%)**	**>65 (%)**	**Negative/Tested (N/N)**	**Baseline IgG Levels (mean ± SD)**	**N (%)**	**Days from COVID-19 to Vaccination (mean ± SD)**
Total	285	157 (55.1)	128 (44.9)	218 (76.5)	68 (23.8)	67/67	0.045 ± 0.064	47 (16.4)	157.5 ± 94.7
BNT-162b2	100 (35)	64 (64)	36 (36)	91 (91)	9 (9)	43/43	0.043 ± 0.044	17 (17)	153.3 ± 93.3
BBIBP-CorV	148 (51.9)	75 (50.6)	73 (49)	91 (61.5)	57 (38.5)	18/18	0.06 ± 0.01	20 (13.5)	131.4 ± 87.2
ChAdOx1-S	25 (8.7)	12 (48)	13 (52)	23 (92)	2 (8)	4/4	0.015 ± 0.015	7 (28)	242.4 ± 94.3
Gam-COVID-Vac	12 (4.2)	6 (50)	6 (50)	12 (100)	0	2/2	0.023 ± 0.016	3 (25)	156.7 ± 73.9

**Table 2:** Detection of specific IgM antibodies at 6 weeks and 3 months after vaccination and after COVID-19 infection.

	BNT-162b2	BBIBP-CorV	ChAdOx1-S	Gam-COVID-Vac	NI	*p*
6 weeks N (%)	34/82 (41.4)	19/95 (20)	3/16 (18.7)	0/10	66/90 (73.3)	<0.001 *
3 months N (%)	3/55 (5.4)	3/53 (5.7)	NA	NA	41/93 (44.1)	<0.001 *

* each vaccinal group vs. NI; NI—naturally immunized, NA—not applicable.

## Data Availability

Data available on request from the corresponding author; the data are not publicly available due to privacy and ethical restrictions.
